# Porcine Interleukin-17 and 22 Co-Expressed by *Yarrowia lipolytica* Enhance Immunity and Increase Protection against Bacterial Challenge in Mice and Piglets

**DOI:** 10.3390/biology11121747

**Published:** 2022-11-30

**Authors:** Junjie Peng, Fang Yang, Jianlin Chen, Shaohua Guo, Linhan Zhang, Dinghao Deng, Jiangling Li, Xuebin Lv, Rong Gao

**Affiliations:** 1 College of Life Science, Sichuan University, Chengdu 610065, China; 2 New Drug Pre-Production Key Laboratory of Sichuan Province, Chengdu 610093, China; 3 School of Laboratory Medicine, Collaborative Innovation Center of Sichuan for Elderly Care and Health, Chengdu Medical College, Chengdu 610500, China; 4 Sichuan Animal Science Academy, Chengdu 610066, China

**Keywords:** pig interleukin-17, interleukin-22, *Yarrowia lipolytica*, immunopotentiator, bacterial infection, immunity

## Abstract

**Simple Summary:**

The abuse of antibiotics leads to the drug resistance of pathogenic bacteria, which greatly threatens the safety of humans and animals. Novel immunoregulatory approaches with safety and effectiveness that promote antimicrobial function and enhance immune response, thus, should be developed as replacements for antibiotics. A recombinant yeast that can express two functional molecules (pig IL-17 and IL-22) was constructed and designated as Po1h-pINA1297-IL-17/22. To evaluate the immunoregulatory activities of the recombinant yeast, Po1h-pINA1297-IL-17/22 was orally fed to 4-week-old female BALB/c mice and then challenged with virulent Salmonella typhimurium. Mucosal, cellular, and humoral immunity were determined by measuring immunoglobulin levels, concentration of cytokines, and the counts of T lymphocytes. The results showed that Po1h-pINA1297-IL-17/22 treated mice had stronger immune responses and resistance to bacterial infection. The subsequent oral inoculation of piglets with Po1h-pINA1297-IL-17/22 confirmed that Po1h-pINA1297-IL-17/22 obviously improved the growth performance and systemic immunity of piglets. The results above indicated that Po1h-pINA1297-IL17/22 could be developed as a promising immunopotentiator to prevent bacterial infections by promotion of immune responses.

**Abstract:**

Drug resistance in economic animals to pathogens is a matter of widespread concern due to abuse of antibiotics. In order to develop a safe and economical immunopotentiator to raise the immunity and antibacterial response as a replacement for antibiotics, a recombinant yeast co-expressing pig interleukin-17 (IL-17) and IL-22 was constructed and designated as Po1h-pINA1297-IL-17/22. To evaluate the immunoregulator activities of Po1h-pINA1297-IL-17/22, two experiment groups (oral inoculation with Po1h-pINA1297 or Po1h-pINA1297-IL-17/22) and a negative control group (PBS) were set up using 4-week-old female BALB/c mice (10/group). The level of cytokines, including IL-2, IL-4, IL-10, and IFN-γ, were detected by ELISA, and the circulating CD4+ and CD8+ lymphocytes were quantified by flow cytometry. The IgG and secretory IgA (SIgA) levels in both small intestine and fecal matter were also measured by ELISA. The results indicated that the IgG antibody titer and SIgA concentration increased significantly in the Po1h-pINA1297-IL17/22 group in comparison with the controls (*p* < 0.05) and so did the cytokine levels in the serum (IL-2, IL-4, IL-10, and IFN-γ). In addition, CD4+ and CD8+ T cells were also obviously elevated in the Po1h-pINA1297-IL17/22 group on 35th day (*p* < 0.05). After challenge with pathogenic *Salmonella typhimurium*, the Po1h-pINA1297-IL17/22 group showed a relatively higher survival rate without obvious infectious symptoms. On the contrary, the mortality of control group reached 80% due to bacterial infection. As for the piglet experiment, 30 healthy 7-day piglets were similarly attributed into three groups. The oral inoculation of piglets with Po1h-pINA1297-IL17/22 also markedly improved the growth performance and systemic immunity (up-regulations of IL-4, IL-6, IL-15, IL-17, IL-22, and IL-23). Overall, the results indicated that Po1h-pINA1297-IL17/22 effectively promoted the humoral and cellular immunity against bacterial infection. These proved the promising potential of Po1h-pINA1297-IL-17/22 to be a potent immunopotentiator for the prevention of microbial pathogen infections.

## 1. Introduction

Antimicrobial resistance (AMR) continues to be an increasing significant danger to mankind and animals, diminishing the capacity to fight against microbial infections and raising the risk of morbidity and death associated with resistant microorganisms [1,2]. Eighty percent of antimicrobial agents generated in the United States are utilized in animal production, while more than seventy percent of antimicrobials produced worldwide are used in food-animal production [3,4]. However, antibiotics were prohibited from promoting animals’ growth in over 86 nations and areas throughout the world by 2018; similarly, antibiotics as feed additives were scheduled to be banned in China by the end of 2020 [5,6]. Therefore, there is an urgent need for the replacement of antibiotics in order to overcome difficult infections in animals and to enhance their antibacterial defense via improving the intestinal microenvironment and mucosal immunity. To reduce the spread of the caused drug resistance, transforming the mechanism of action of feed additives, such as triggering T helper cells to participate more effectively to optimize the quality and durability of antibody responses or inducing effector CD4+ and CD8+ T cells to eliminate intracellular pathogens, would potentially be finer approaches [7].

Cytokines have been utilized to be more potent immunomodulators with efficient effects on the immune system [8]. IL-17A containing 462 bp coding deduced 153 amino acid residues (AA) belongs to the IL-17 cytokine family [9] and has an important role in the host protection against specific pathogens, such as F4+ ETEC, Candida albicans, Citrobacter rodentium, Salmonella typhimurium, Giardia muris, and H5N1 influenza virus [10,11,12,13,14,15,16,17]. As a member of the IL-10 cytokine family, IL-22 serves as a comprehensive mediator that plays important roles in regulating the innate immune response, repairing tissue damage, and stimulating the regeneration of them [18]. By producing antimicrobial proteins (AMPs), IL-22 secreted from mucin and differentiated via goblet cells [19,20,21], provides essential barrier functions that protect the gut from infection by external pathogens [22].

Our previous experiments proved that pig IL-4, IL6, and fused IL-4/6 genes can remarkably boost the immune system of mice [23]. The fused gene of CAMPs and IL-4/6 can enhance the resistance of mice against *Staphylococcus aureus* and *Escherichia coli* [24]. In order to verify the potential of co-expression of IL-17 and IL-22 as an immunomodulator, recombinant *Yarrowia lipolytica* was utilized in this study to investigate its impact on the immunity of mice [25]. *Yarrowia lipolytica* was chosen as the expression host in view of the exceptional safety and eco-friendliness.

## 2. Materials and Methods

### 2.1. Bacteria and Plasmids

The *Yarrowia lipolytica* Po1h strain (MatA, ura3-202, xpr2-322, axp1-2, Ura3-, ∆AEP, ∆AXP, Suc+), kindly provided by Professor Catherine Madzak from INRAE French National Institute for Research on Agriculture Food and Environment, was used as the host. The plasmid pINA1297, an *E. coli*–*Lipolytica* shuttle plasmid, kindly provided by Professor Catherine Madzak, was replicated in *E. coli* DH5α and then utilized to transfer the foreign gene into the Po1h strain.

### 2.2. Construction of Recombinant Yarrowia lipolytica pINA1297-IL-17/22

The sequences for porcine IL-17 and IL-22 were obtained separately from GenBank (Accession No. NM_001005729.1 and No. KX588234.1). The coding sequences were optimized to adapt the codon preference of *Yarrowia lipolytica* and to promote the translation of the recombinant gene. The fusion gene was designated as rIL-17/22 (XPR2 pre-IL-17-GSG-P2A-XPR2 pre-IL-22), the signal peptide sequence XPR2 pre and the linker gene fragment (GSG-P2A) were used to achieve co-expression of pig IL-17 and IL-22, and its size was 1086 base pairs (bp). The rIL-17/22 gene was fused by Beijing Tsingke Biotechnology Company (Chengdu, China), and then, it was ligated into pINA1297. The ligation fragment was transformed into DH5α *E. coli* competent cells and cultured on a Luria Bertani (LB) agar plate with 50 μg/mL kanamycin (Kana). The positive pINA1297-IL-17/22 clones were screened out and sequenced by the Youkang Biotechnology Company (Chengdu, China). The recombinant pINA1297-IL-17/22 and the wide-type plasmid pINA1297 were digested with restriction endonuclease *Not* I, and then the target gene fragments were transformed into the competent Po1h strain by Frozen-EZ Yeast Transformation Ⅱ Kit™ (The Epigenetics COMPANY, USA), with the resulting cells named Po1h-pINA1297-IL-17/22 and Po1h-pINA1297, respectively. The colonies were able to grow on an MD plate. The presence of pINA1297-IL-17/22 in the Po1h cells was confirmed by PCR and agarose gel electrophoresis. The primers for the pINA1297 are listed as: forward primer: Pc-F GAGCGTTTGCCAGCCACAGATTTTC; reverse primer: Pc-R CATTGATGGACAGGTAGTAGGAGGC.

### 2.3. Analysis of rIL-17/22 Expression by ELISA and Western Blotting

The supernatant was collected by centrifugation and detected with pig interleukin 17 (IL-17) and 22 (IL-22) ELISA Kit (CUSABIO, Wuhan, China). Briefly, 5 µL of the protein sample was added to 1 µL 6× loading buffer and then analyzed by 10% sodium dodecyl sulfate–polyacrylamide gel electrophoresis (SDS-PAGE) and Western blotting. The rabbit anti-pig IL-17 and IL-22 McAb (Abmart Ltd., Shanghai, China) and the horseradish peroxidase (HRP)-labeled goat anti-rabbit immunoglobulin G with heavy chain and light chain [IgG (H + L)] (Abmart Ltd., Shanghai, China) were used as the primary and secondary antibody, respectively. M5 HiPer ECL Western HRP Substrate (Mei5 Biotech Co., Ltd., Beijing, China) was then employed to detect the specific ban via the Bio-Rad ChemiDoc Touch system (Bio-Rad, Berkeley, CA, USA).

### 2.4. Bioactivity Assay of rIL-17/22 In Vitro

Tibetan pig’s PBMCs were isolated by a lymphocyte separation kit (Dakewe Biotech Co., Ltd., Shenzhen, China) and incubated with 5 µg/mL Con A (Sigma Chemical Co., St. Louis, MO, USA) in 1640 medium for 24 h. After harvesting and washing three times by centrifugation, the lymphoblast cells were then cultured in 1640 medium in 6 × 10^6^/mL. The treated cells were added into a 96-well plate (50 µL/well), along with the same supernatant from transformed Po1h. The cells were incubated at 37 °C in a 5% CO_2_ atmosphere for 48 h. Then, in each well of the cell counting kit (CCK)-8, 100 µL 2-(2-methoxy-4-nitrophenyl)-3-(4-nitrophenyl)-5-(2,4-disulfophenyl)-2H-tetrazolium sodium salt (WST)-8 was added, and the cells were cultured for another 2 h under the same condition. OD450 was detected by Microtiter plate Reader 680 (Bio-Rad, Hercules, CA, USA).

### 2.5. Preparation of Recombinant Yeast

Recombinant Po1h transformants were grown on YPD plates (10 g/L yeast extract, 20 g/L tryptone, 20 g/L glucose, and 15 g/L agar) at 28 °C for 48 h. A single colony then was inoculated into liquid YPD medium (10 g/L yeast extract, 20 g/L tryptone, and 20 g/L glucose) and cultivated at 28 °C and 200 rpm respectively. The overnight cultures (100 mL) were inoculated into fresh 1 L of liquid YPD medium at a 10% inoculation ratio, in 2 L flasks, and cultured at 28 °C and 200 rpm for 2 days.

### 2.6. Experimental Protocol for Mice

Specific-pathogen-free (SPF) BALB/c female mice were provided by Laboratory animal center, Sichuan university (Chengdu, China). Thirty-nine 4 week old mice were randomly separated into three groups, designed as the treatment group Po1h-pINA1297-IL-17/22 and the control groups Po1h-pINA1297 and PBS. Mice in the Po1h-pINA1297-IL-17/22, Po1h-pINA1297, and PBS groups were orally inoculated with the fresh Po1h-pINA1297-IL-17/22 strain (4.0 × 10^8^ colony forming unit-CFU), Po1h-pINA1297 (4.0 × 10^8^ CFU) and 100 µL PBS, respectively, every three days. The first inoculation was performed on day 0, and a total of 12 inoculations were conducted during the 35-day period. The weight of every mouse was recorded weekly for 5 weeks to evaluate growth performance. The EDTA-K2 blood samples (100 µL each) were collected from the tail veins of mice on the 7th, 14th, 21st, 28th, and 35th day post the oral inoculation. On the 28th day post inoculation, three mice of each group were randomly euthanized to collect feces and small intestines to detect the SIgA concentration in mucosal tissues. Additionally, on the 35th day post the inoculation, ten mice in each group, (Po1h-pINA1297-IL-17/22, Po1h-pINA1297, and PBS) were orally challenged with 0.3 mL 1.0 × 10^10^ CFU *Salmonella typhimurium* (ATCC14028). The challenged mice were observed daily for 14 days. After 14 days observation, all the remained mice were euthanized to collect feces and small intestines for SIgA detection and morphological analysis. The parts of small intestinal tissues were soaked in a 10% formalin solution, then sent to Chengdu Lilai Biotechnology Co., Ltd. (Chengdu, China) for further staining with Hematoxylin and eosin (H & E) and measurements. The Animal Ethics Committee (ACE) of Sichuan University authorized the animal experiments (license: SYXK-Chuan-2019-283). All protocols and animal welfare standards exactly adhered to the animal management regulations of Sichuan University.

### 2.7. Animal Experimental Protocol for Piglets

Thirty healthy 7-day piglets (hybrids of Duroc and Tibetan pig) were provided by Sichuan Animal Science Academy. Diagnosed by ELISA and RT-PCR, all piglets were negative for PCV-2, PRRSV, CSFV, and mycoplasma pneumonia. The piglets were randomly divided into three groups (Po1h-pINA1297, Po1h-pINA1297-IL-17/22 and PBS). Piglets in Po1h-pINA1297 and Po1h-pINA1297-IL-17/22 groups received the fresh respective strains (1.0 × 10^9^ CFU each) orally every three days during the 56-day experiment (a total of 18 inoculations, started at the age of 7 days, day 0 post inoculation). At the same time, the PBS group received the same volume PBS solution as the blank control. Then, 5 mL EDTA-stabilized precaval venous blood sample was collected from each anesthetized piglet on days 14, 28, 42 and 56 after inoculation to assay immunological changes (cytokine levels). The initial and final weight of the experimental pigs in each group were recorded to evaluate the treatment effect on animal growth performance. All pigs were fed under the same condition. The care and use of the experimental animals complied with Chinese animal welfare laws, guidelines, and regulations.

### 2.8. Evaluation of the Immunoregulator Effect of Po1h-pINA1297-IL-17/22 In Vivo

#### 2.8.1. Assay of Cytokines in the Serum of Mice by ELISA

On the 7th, 14th, 21st, 28th, and 35th days post the inoculation, a 100 µL blood sample was taken from each mouse to identify the cytokine levels (IL-2, IL-4, IL-6, IL-10, and IFN-γ) in the serum by the sandwich ELISA kit (sELISA, SinoBestBio, Shanghai, China).

#### 2.8.2. Cytokine Levels in the Plasma of Piglets by ELISA

The cytokine levels (pig IL-4, IL-6, IL-15, IL-23, IL-17 and IL-22) in the plasma were measured by commercial ELISA kits (Cusabio Biotech Co., Ltd., Hubei, China and SinoBestBio, Shanghai, China) following the manufacturer’s instructions on days 14, 28, 42, and 56 after inoculation. The concentrations of cytokines were calculated according to the standard curve supplied in the kits.

#### 2.8.3. Changes of CD4+ and CD8+ T Cells in the Blood by Flow Cytometry (FCM)

Anti-mouse CD3e with fluorescein isothiocyanate (FITC), anti-mouse CD8a with phycoerythrin (PE), and anti-mouse CD4 antibodies with PerCP-Cy5.5 were obtained from eBioscience, USA. Each test consisted of 50 µL of peripheral blood sample on days 0 and 35 post inoculation, the first and 12th inoculation, 0.5 µL anti-mouse CD3e, 0.25 µL anti-mouse CD8a, and 0.25 µL anti-mouse CD4. After 20 min incubation in the dark, 2 mL lysing solution (Becton Dickinson, East Rutherford, NJ, USA, 10%, *v*/*v*) was mixed to lyse erythrocytes thoroughly. The surviving cells were washed twice with PBS and centrifuged for 5 min at 500× *g* between each step. FCM analysis was performed with a FACScan flow cytometer (BD Biosciences, Franklin Lakes, NJ, USA) as the instruction.

#### 2.8.4. Measures of IgG in Serum and SIgA in Small Intestines and Feces by ELISA

The changes in immunoglobulins in sera were detected on days 14th, 21st, 28th, and 35th post inoculation by a Mouse IgG quantitation ELISA Kit (ebioscience, San Diego, CA, USA) following the manufacturer’s protocols. First, 1-cm-long fresh small intestines were washed with 500 µL PBS containing protease inhibitor and centrifuged at 12,000× *g* for 20 min at 4 °C to remove debris before storage at −80 °C. The IgA sELISA kit (SinoBestBio, Shanghai, China) was employed to analyze the total SIgA in the lavage fluid following the instruction.

### 2.9. Statistical Analysis

GraphPad Prism 9 (GraphPad Software, San Diego, CA, USA) was employed for statistical analysis and graphical presentations. The differences among the groups were conducted by one-way ANOVA and Tukey’s test for multiple comparisons, If *p* < 0.05, the difference is significant. All data were analyzed within each sampling time point.

## 3. Results

### 3.1. Construction of the Po1h-pINA1297-IL-17/22

**Figure 1:**
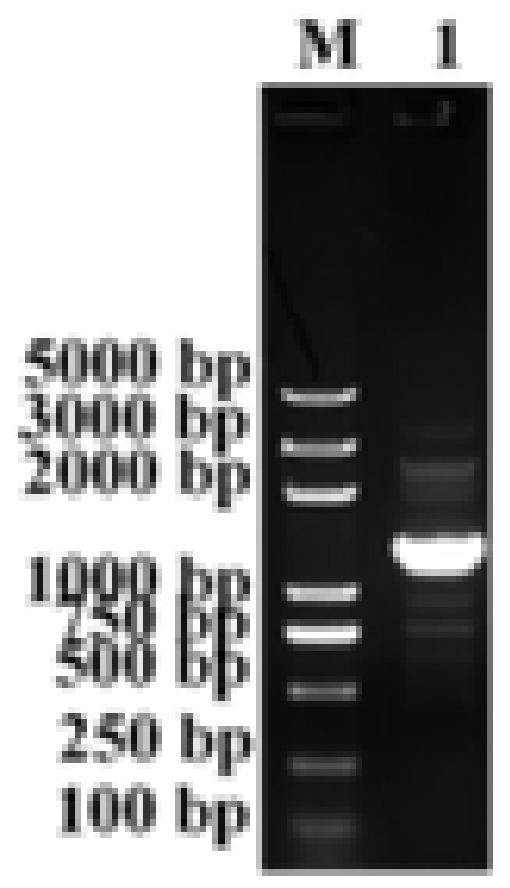
The construction of the Po1h-pINA1297-IL-17/22 strain. Analysis of the *rIL-17/22* gene in Po1h by PCR. Lane M: DL 5000 DNA marker; Lane 1: Po1h-pINA1297-IL-17/22.

As shown in Figure 1, the rIL-17/22 fusion gene was found in the Po1h strain.

### 3.2. Expression of the Po1h-pINA1297-IL-17/22

**Figure 2:**
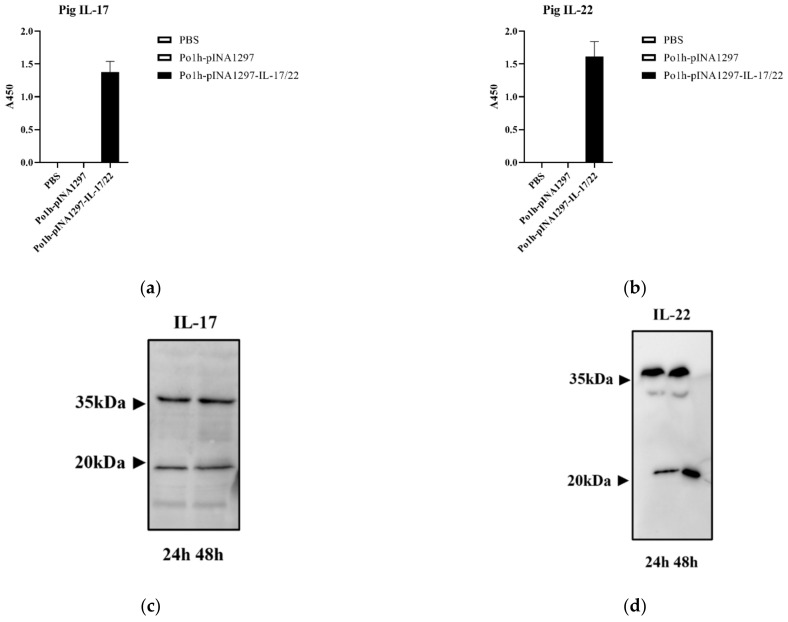
The expression analysis of Po1h-pINA1297-IL-17/22. Expression analysis of rIL-17 and rIL-22 in Po1h by ELISA (**a**,**b**) and by Western blot (**c**,**d**).

Figure 2a,b showed the mass of pig IL-17 and IL-22 was 1.432 µg/L and 1.631 µg/L, respectively, in the supernatant. Western blot results are shown in Figure 2c,d, protein bands of approximately 20 KDa match with predicted sizes of rIL-17 (17.3 KDa) and rIL-22 (23.01 KDa). These indicate that recombinant pig IL-17 and IL-22 were successfully co-expressed in Po1h-pINA1297-IL-17/22.

### 3.3. Bioactivity of the Po1h-pINA1297-IL-17/22

**Figure 3:**
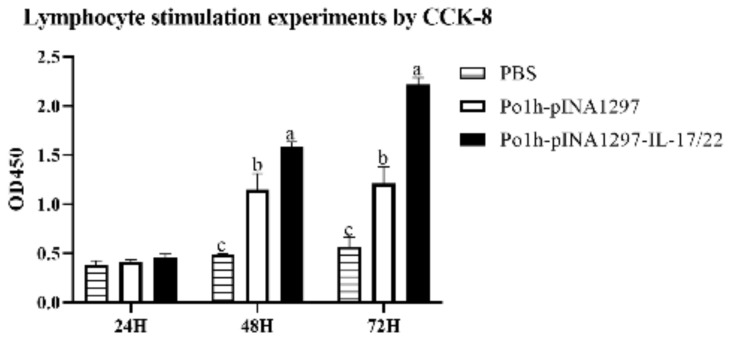
Cell viability analysis via CCK8. The data with different lowercase letter are significantly different, *p* < 0.05, and the following are the same as here.

It is obvious from Figure 3 that the interleukins expressed by Po1h provoked remarkable lymphocyte activation and proliferation in comparison with the controls (*p* < 0.05).

### 3.4. Weight Change in the Experimental Mice

By the end of the 35th day post inoculation, the mice in the Po1h-pINA1297-IL-17/22 group gained more weight than the control groups (Table 1), as determined using an unpaired *t*-test (*p* < 0.05), though there was no significant difference among the three groups regarding the initial and end weights of mice. It indicated that the oral inoculation of Po1h-pINA1297-IL-17/22 resulted in a marked weight gain effect.

### 3.5. Evaluation of the Immunoregulator Activities of Po1h-pINA1297-IL-17/22 in Mice

#### 3.5.1. Analysis of Total IgG

**Figure 4:**
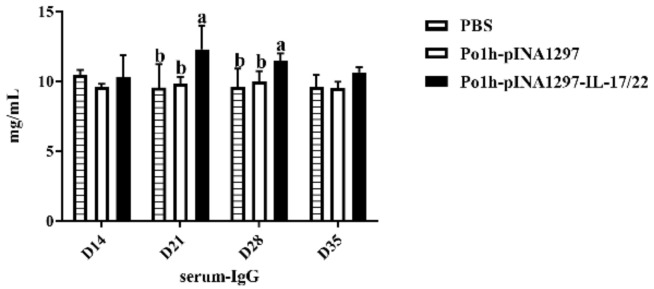
Changes in IgG levels in the serum of the experimental mice. The data with different lowercase letter are significantly different, *p* < 0.05).

On the 21st and 28th day post inoculation, the 7th and 8th inoculations, the IgG content in the Po1h-pINA1297-IL-17/22 group was significantly higher than in the PBS and Po1h-pINA1297 (*p* < 0.05) groups (Figure 4). Furthermore, on the 21st day post inoculation, the IgG level reached the peak in the Po1h-pINA1297-IL-17/22 group.

#### 3.5.2. Changes of Cytokines Levels

**Figure 5:**
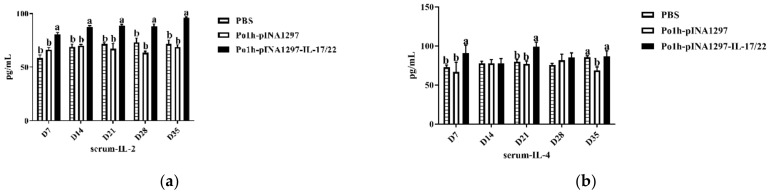

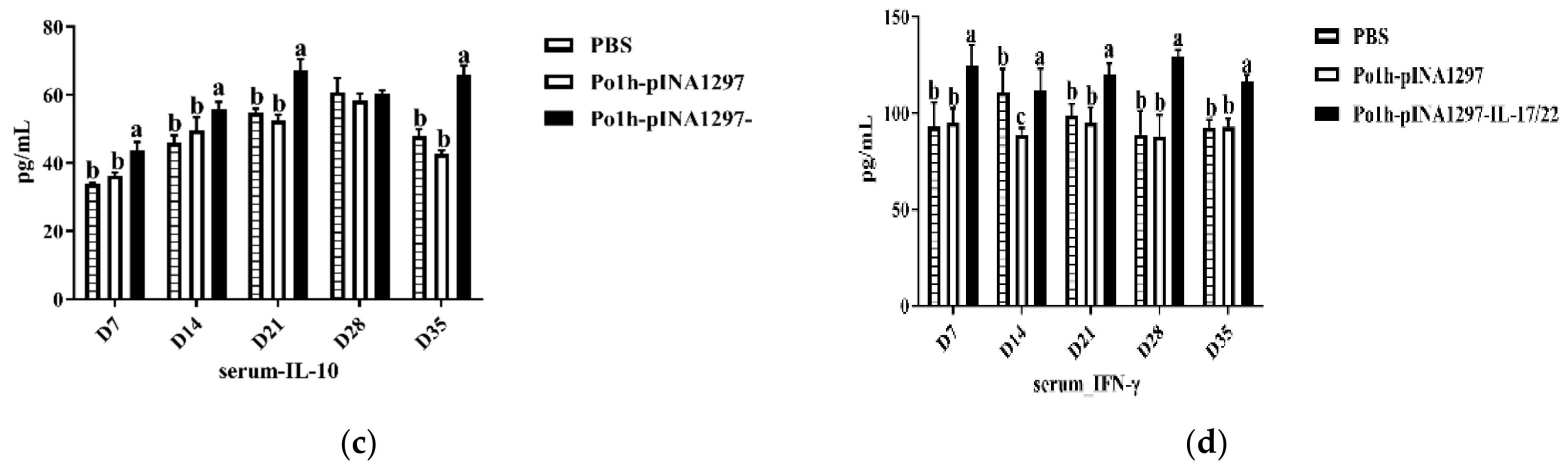
Concentrations of IL-2 (**a**), IL-4 (**b**), IL-10 (**c**), and IFN-γ (**d**) in the serum of the mice post inoculation. The data with different lowercase letter are significantly different, *p* < 0.05.

As shown in Figure 5, the levels of IL-2, IL-10, and IFN-γ (Figure 5a,c,d) of the Po1h-pINA1297-IL-17/22 group were significantly higher than the PBS and Po1h-pINA1297 groups (*p* < 0.05) during the whole observation. Moreover, the concentration of IL-4 (Figure 5b) in the Po1h-pINA1297-IL-17/22 group was significantly higher compared to that from the PBS and Po1h-pINA1297 on the 7th and 21st day post the inoculation, the 3rd and 7th inoculation (*p* < 0.05).

#### 3.5.3. Changes of CD4+ and CD8+ T Cells

**Figure 6:**
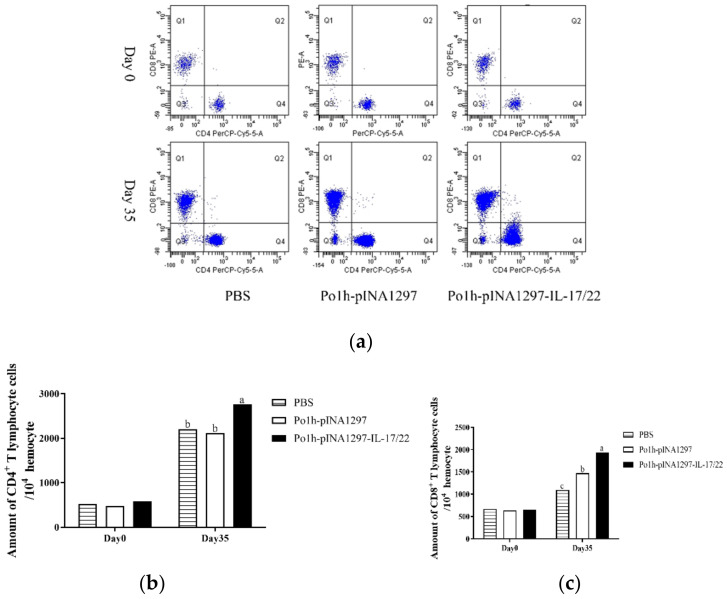
Changes in CD4+ and CD8+ T cells in the peripheral blood of mice on the 35th day post inoculation. (**a**) Changes in the CD4+ and CD8+ T cells. (**b**) The counts of CD4+ T cells. (**c**) The counts of CD8+ T cells. The data with different lowercase letters are significantly different, *p* < 0.05.

The T cell subtypes were analyzed in the blood by FCM. On the 35th day post the inoculation, CD4+ and CD8+ T cells markedly increased in the mice from the Po1h-pINA1297-IL-17/22 group compared to that in the PBS and Po1h-pINA1297 groups (Figure 6b,c) (*p* < 0.05).

#### 3.5.4. Mucosal SIgA in the Gut

**Figure 7:**
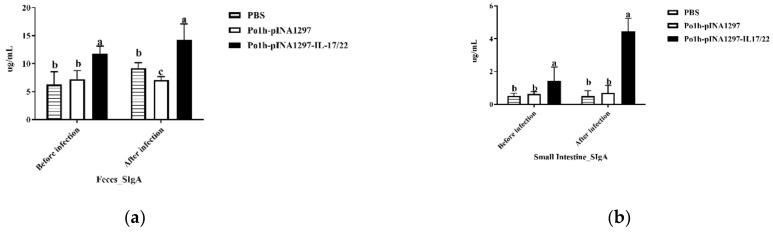
Total SIgA concentrations (**a**,**b**) in the feces and intestines of the mice. The data with different lowercase letters are significantly different, *p* < 0.05.

In the feces, the total SIgA in the Po1h-pINA1297-IL-17/22 group were significantly higher than the PBS and Po1h-pINA1297-IL-17/22 groups (Figure 7a) (*p* < 0.05). In the intestines of the Po1h-pINA1297-IL-17/22 group, the total SIgA was significantly elevated compared to other groups (Figure 7b) (*p* < 0.05). Furthermore, in the PBS and Po1h-pINA1297 groups, the concentrations of total SIgA did not show remarkable difference in either the feces or the intestines.

#### 3.5.5. Evaluation of the Protection against *Salmonella typhimurium* Challenge

**Figure 8:**
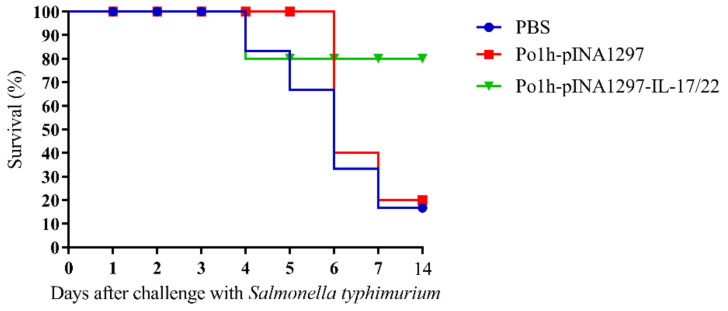
The survival percentage of mice after challenge.

The survival percentage of mice is shown in Figure 8 according to 14 days observation after oral challenge at 35 days post inoculation with tolerance *Salmonella typhimurium*. The group treated with Po1h-pINA1297-IL-17/22 manifested the strongest protection against the infection, which exhibited 80% survival. Furthermore, 80% of the mice in groups PBS and Po1h-pINA1297 were dead after the challenge.

#### 3.5.6. Histological Examination of Small Intestine

**Figure 9:**
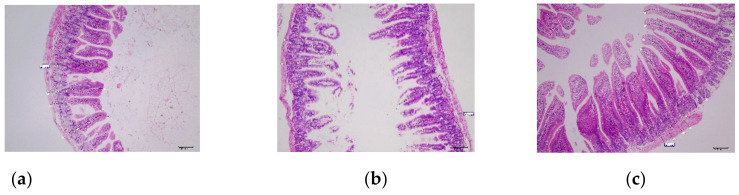

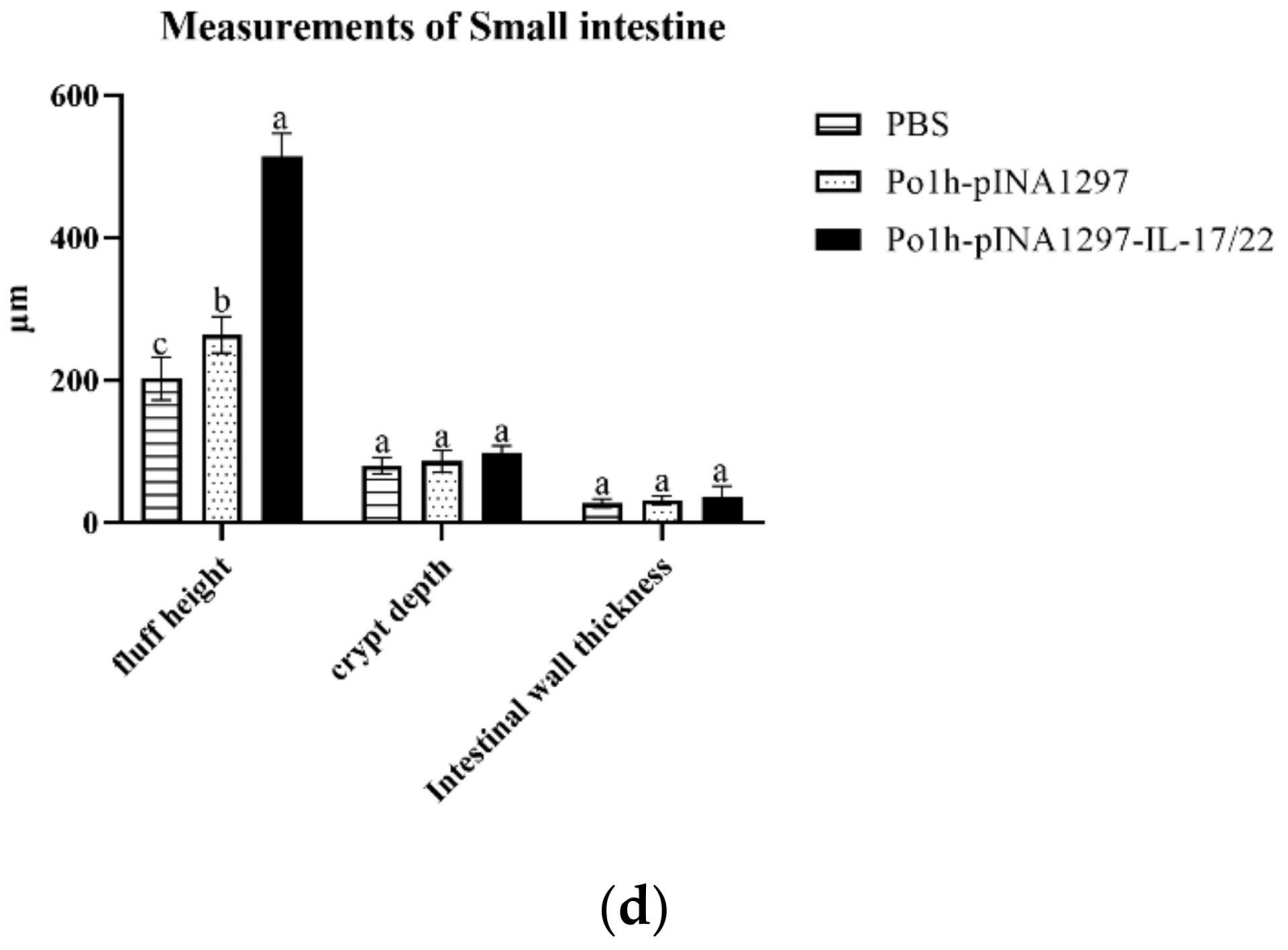
Hematoxylin and eosin staining of small intestine tissues. Histologic examination showed the fluff height, crypt depth, and intestinal wall thickness in PBS (**a**), Po1h-pINA1297 (**b**) and Po1h-pINA1297-IL-17/22 groups (**c**) and their statistical comparison (**d**) respectively. Scale bar = 100 µm. The data with different letter are significantly different, *p* < 0.05.

After 14 days observation, the small intestines of all groups were collected and stained with H & E (a–c). As shown in Figure 9c,d, the fluff height in the Po1h-pINA1297-IL-17/22 group was significantly higher than that in the PBS and Po1h-pINA1297 groups (*p* < 0.05). However, there is no marked difference in crypt depth and intestinal wall thickness between these groups (*p* > 0.05). These results indicated that mice in Po1h-pINA1297-IL-17/22 have a stronger ability to maintain intestinal structure homeostasis.

### 3.6. Weight Change of the Experimental Piglets

**Figure 10:**
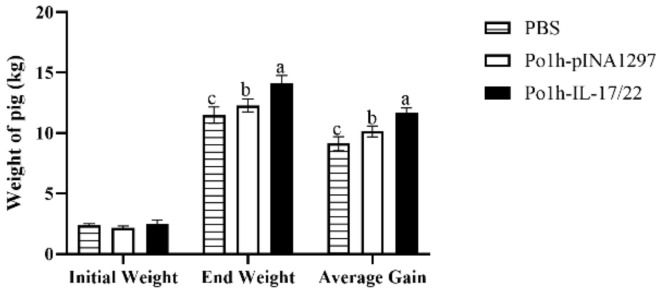
Changes in piglet weights during 8 weeks of observation (n = 10/group). The data with different lowercase letter are significantly different, *p* < 0.05.

Figure 10 demonstrates that the weight gain in the Po1h-pINA1297-IL-17/22 group was significantly higher than the control piglets (*p* < 0.05), and no piglets were dead during the experiment. This indicates that the oral inoculation with Po1h-pINA1297-IL-17/22 can improve the growth performance of piglets.

### 3.7. Evaluation of Immunoregulator Activities of Po1h-pINA1297-IL-17/22 in Pig

#### 3.7.1. Change of Cytokine Levels in Plasma

**Figure 11:**
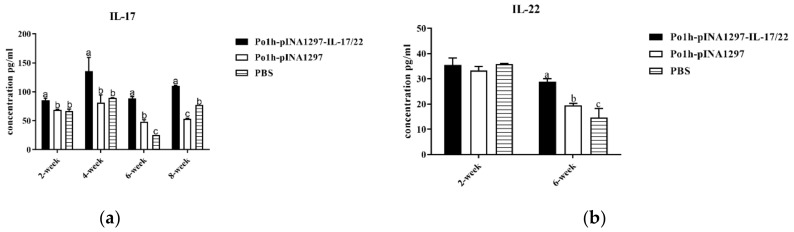

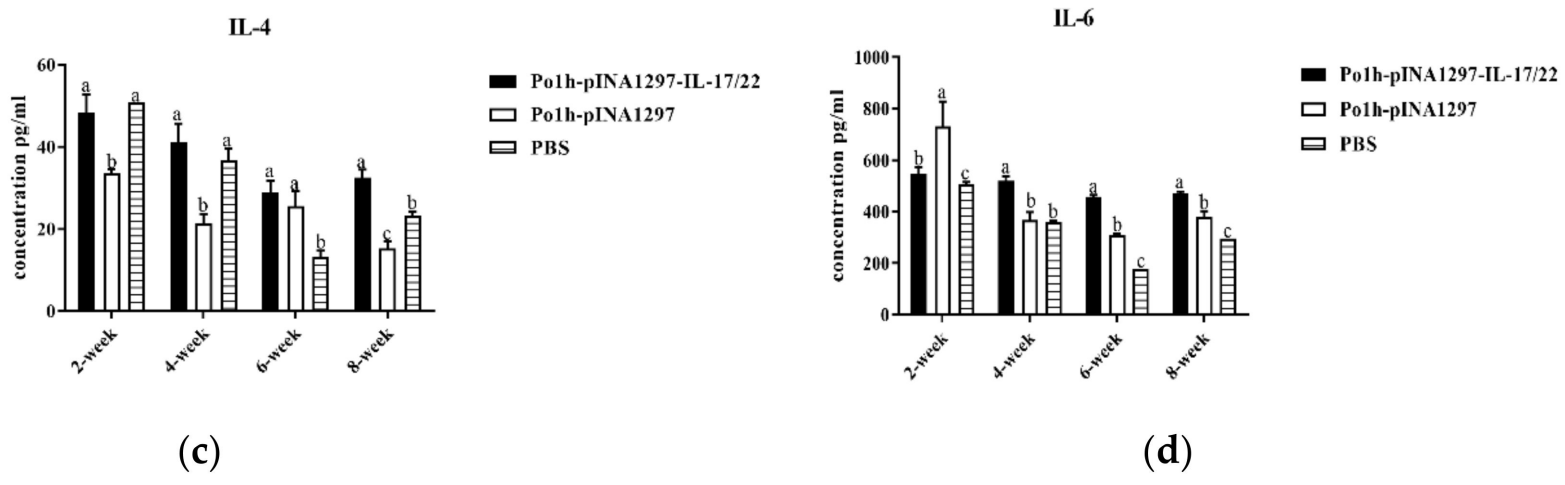
Change in IL-17 (**a**), IL-22 (**b**), IL-4 (**c**) and IL-6 (**d**) levels in the plasma of the piglets. The data with different lowercase letters are significantly different, *p* < 0.05.

As shown in Figure 11, the concentration of IL-17 (Figure 11a) in the Po1h-pINA1297-IL-17/22 group were significantly higher than that in the PBS and Po1h-pINA1297 groups (*p* < 0.05) during the whole observation. Moreover, the concentration of IL-22 (Figure 11b) in the Po1h-pINA1297-IL-17/22 group was significantly higher compared to that from the PBS and Po1h-pINA1297 (*p* < 0.05) on the 42nd day post the inoculation (*p* < 0.05).

The IL-4 and IL-6 of the Po1h-pINA1297-IL-17/22 mice significantly increased compared to those of the PBS and Polh1297 mice from 14th to 56th days after inoculation (Figure 11c,d) (*p* < 0.05).

#### 3.7.2. Change in Immune Memory Relative Cytokine Levels in Plasma

**Figure 12:**
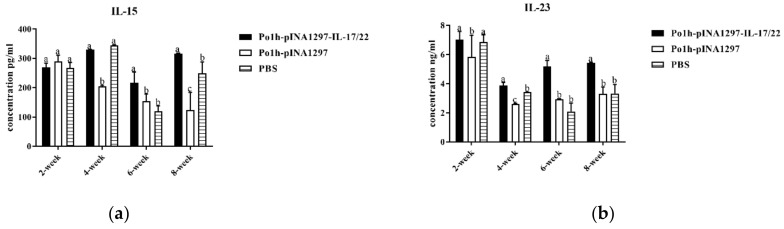
Changes in the IL-15 (**a**) and IL-23 (**b**) levels in the plasma of the piglets.

The levels of IL-15 and IL-23 in the Po1h-pINA1297-IL-17/22 group were significantly higher compared to the PBS and Po1h-pINA1297 group on the 42nd and 56th day post inoculation (Figure 12a,b) (*p* < 0.05).

## 4. Discussion

The abuse of antibiotics continues to exacerbate the antibiotics resistance crisis; hence, there is an urgent need for a substitute. Cytokines as immunoregulators have been widely studied due to their vital roles that synergistically modulate the immune system [26]; for instance, IL-17 has an important role in the host protection against specific pathogens such as F4+ ETEC et al. [12]. IL-22 has been reported to act by inducing barrier protection against pathogens through goblet cell differentiation and mucin secretion [19,20,21], production of anti-microbial proteins (AMPs) [22]. Considering the advantages of these two cytokines, the synergic effects of IL-17 and IL-22 on the immune response of mice and their anti-infectious capacity were explored in this study.

We first developed the recombinant *Yarrowia. lipolytica* Po1h-pINA1297-IL-17/22 strain as an oral agent that expressed bioactive IL-17/22 in vitro. We then assessed the expression and bioactivity of Po1h-pINA1297-IL-17/22 in animals. The 2A self-cleavage sequence was recruited to link IL-17 and IL-22 to produce two single proteins. This genetical engineering way ensured the intact bioactivity of IL-17 and IL-22, which was confirmed by Western blot, ELISA and lymphocytes proliferation in vitro. Since the 2A self-cleaving efficiency is not 100% [27], a protein band of approximately 40 KDa (fused IL-17 and IL-22) appears in Western blot.

In our animal experiments, the mice in the Po1h-pINA1297-IL-17/22 group displayed an evident promotion of growth compared to the control groups. Our previous works also found similar results that fusion protein IL-4/6 engineered by the same technique or single IL-23 and IL-17B both improved the animals’ growth performance [28,29,30]. The results indicate that Po1h-pINA1297-IL-17/22 has the effect of increasing body weight gain. We also observed up-regulations of IL-2, IL-4, IL-10, and IFN-γ in the Po1h-pINA1297-IL-17/22 group. IL-2 and IFN-γ can boost Th1 cell-type immune responses to attack pathogens inside cells. IL-4 can enhance humoral immune responses, such as the production, class switching and secretion of immunoglobulin [31,32]. Th2 cells generate IL-4 and IL-10, which promote the differentiation of B cells to produce IgA and IgG and the proliferation of monocytes to involve in the immune defense [33]. The cytokine levels are closely correlated with the increase in CD4+ and CD8+ T lymphocytes and IgG levels. Taken together, these results confirm that Po1h-pINA1297-IL-17/22 potentiates Th2-type immunity—the antibody response and the Th1 cellular immunity against infection [34].

The SIgA antibody is the major index of local humoral immunity [35]. Both T cell-dependent and T cell-independent pathways can induce SIgA responses [36]. Recently, IL-17A has been implicated in the production of SIgA directed against gut-dwelling pathogens [37,38]. During the 35-day period, Po1h-pINA1297-IL-17/22 provoked a significant increase in SIgA compared to the control mice. The increases in total SIgA in the feces and intestines suggested that the Po1h-pINA1297-IL-17/22 elevated the mucosal humoral immunity, which would facilitate the strong resistance against pathogenic bacteria in the gut. The results indicated that Po1h-pINA1297-IL-17/22 can enhance the humoral and mucosal immunity.

The *Salmonella typhimurium* challenge result showed the strong protective effect of the Po1h-pINA1297-IL-17/22. The majority of inoculated mice were protected and did not manifest any symptoms or lesions resulted from the bacterial infection. On the contrary, the challenge bacteria severely infected the control mice, and the majority of them were dead from infection-related diseases within seven days; the rest survived without obvious lesions. Interestingly, it was found that from the H & E staining of the small intestine tissue, the fluff height of Po1h-pINA1297-IL-17/22 mice was significantly higher than that of the PBS and Po1h-pINA1297 groups. It further proved that the mice from the Po1h-pINA1297-IL-17/22 group have stronger resistance against the infection. At the same time, Po1h-pINA1297-IL-17/22 manifested a better enhancement of innate and adaptive immunity, which is mirrored from the stronger humoral and cellular immunity.

As for piglets, the Po1h-pINA1297-IL-17/22 group showed markedly better growth performance in comparison with the control groups for 56 days, and the up-regulations of IL-4, IL-6, IL-15, IL-17, IL-22, and IL-23 were observed in Po1h-pINA1297-IL-17/22, which is accordance with the remarkable increase in weight gain. These results were similar with those of the treated mice. IL-15 is vital for proliferation and maintaining of the memory T-cells pool; It is well known that IL-23 can promote Th17 maturation. The elevated levels of IL-4, IL-6, IL-15, and IL-23 in Po1h-pINA1297-IL-17/22 piglets probably indicated the elevation of the humoral immunity and immune memory function of piglets, which is beneficial for the growth performance and disease resistance of piglets. The exact molecular mechanism for the growth improvement and immunity enhancement of piglets remains unclear and needs more exploration to clarify.

In a word, we first constructed a recombinant *Yarrowia. lipolytica* Po1h-pINA1297-IL-17/22-expressing pig IL-17/22 and also verified that the rIL-17/22 expressed by Po1h-pINA1297-IL-17/22 could up-regulate the local and systemic immune responses of mice. The subsequent animal experiment confirmed that oral inoculation of Po1h-pINA1297-IL-17/22 obviously protected mice from the *Salmonella typhimurium* challenge and improved the growth performance and systemic immunity of piglets. These would inspire a development of promising cost-effective immunopotentiators against infection.

## 5. Conclusions

These results demonstrated that the recombinant Yarrowia. Lipolytica co-expressing pig IL-17/22 was successfully constructed and would facilitate the development of Po1h-pINA1297-IL-17/22 as a potent and economical immunopotentiator for the prevention of animal bacterial infections.

## Figures and Tables

**Table 1:** Changes in mouse weights during 5 weeks of observation (*n* = 10/group).

Group	Initial Weight (g)	End Weight (g)	Average Gain (g)
PBS	18.49 ± 1.07	19.08 ± 1.41	0.59 ± 0.10 ^b^
Po1h-pINA1297	18.53 ± 1.55	19.50 ± 1.80	0.97 ± 0.08 ^b^
Po1h-pINA1297-IL-17/22	17.93 ± 1.45	20.32 ± 1.49	2.39 ± 0.19 ^a^

One-way ANOVA and Tukey’s test was adapted for statistical analysis. The weights are listed as mean ± SEM. *p* < 0.05. The data with a different lowercase letter are significantly different, *p* < 0.05.

## Data Availability

Not applicable.
